# Human health implications of organic food and organic agriculture: a comprehensive review

**DOI:** 10.1186/s12940-017-0315-4

**Published:** 2017-10-27

**Authors:** Axel Mie, Helle Raun Andersen, Stefan Gunnarsson, Johannes Kahl, Emmanuelle Kesse-Guyot, Ewa Rembiałkowska, Gianluca Quaglio, Philippe Grandjean

**Affiliations:** 1 Karolinska Institutet, Department of Clinical Science and Education, Södersjukhuset, 11883 Stockholm, Sweden; 2 Swedish University of Agricultural Sciences (SLU), Centre for Organic Food and Farming (EPOK), Ultuna, Sweden; 3 University of Southern Denmark, Department of Public Health, Odense, Denmark; 4 Swedish University of Agricultural Sciences (SLU), Department of Animal Environment and Health, Skara, Sweden; 5 University of Copenhagen, Department of Nutrition, Exercise and Sports, Frederiksberg, Denmark; 6 Research Unit on Nutritional Epidemiology (U1153 Inserm, U1125 INRA, CNAM, Université Paris 13), Centre of Research in Epidemiology and Statistics Sorbonne Paris Cité, Bobigny, France; 7 Warsaw University of Life Sciences, Department of Functional & Organic Food & Commodities, Warsaw, Poland; 8 Scientific Foresight Unit (Science and Technology Options Assessment [STOA]), Directorate-General for Parliamentary Research Services (EPRS), European Parliament, Brussels, Belgium; 9 Harvard T.H. Chan School of Public Health, Department of Environmental Health, Boston, USA

**Keywords:** Agricultural crops, Antibiotic resistance, Food safety, Nutrients, Organic food, Pesticide residues

## Abstract

This review summarises existing evidence on the impact of organic food on human health. It compares organic vs. conventional food production with respect to parameters important to human health and discusses the potential impact of organic management practices with an emphasis on EU conditions. Organic food consumption may reduce the risk of allergic disease and of overweight and obesity, but the evidence is not conclusive due to likely residual confounding, as consumers of organic food tend to have healthier lifestyles overall. However, animal experiments suggest that identically composed feed from organic or conventional production impacts in different ways on growth and development. In organic agriculture, the use of pesticides is restricted, while residues in conventional fruits and vegetables constitute the main source of human pesticide exposures. Epidemiological studies have reported adverse effects of certain pesticides on children’s cognitive development at current levels of exposure, but these data have so far not been applied in formal risk assessments of individual pesticides. Differences in the composition between organic and conventional crops are limited, such as a modestly higher content of phenolic compounds in organic fruit and vegetables, and likely also a lower content of cadmium in organic cereal crops. Organic dairy products, and perhaps also meats, have a higher content of omega-3 fatty acids compared to conventional products. However, these differences are likely of marginal nutritional significance. Of greater concern is the prevalent use of antibiotics in conventional animal production as a key driver of antibiotic resistance in society; antibiotic use is less intensive in organic production. Overall, this review emphasises several documented and likely human health benefits associated with organic food production, and application of such production methods is likely to be beneficial within conventional agriculture, e.g., in integrated pest management.

## Background

The long-term goal of developing sustainable food systems is considered a high priority by several intergovernmental organisations [1–3]. Different agricultural management systems may have an impact on the sustainability of food systems, as they may affect human health as well as animal wellbeing, food security and environmental sustainability. In this paper, we review the available evidence on links between farming system (conventional vs organic) and human health.

Food production methods are not always easy to classify. This complexity stems from not only the number and varying forms of conventional and organic agricultural systems but also resulting from the overlap of these systems. In this paper, we use the term “conventional agriculture” as the predominant type of intensive agriculture in the European Union (EU), typically with high inputs of synthetic pesticides and mineral fertilisers, and a high proportion of conventionally-produced concentrate feed in animal production. Conversely, “organic agriculture” is in accordance with EU regulations or similar standards for organic production, comprising the use of organic fertilisers such as farmyard and green manure, a predominant reliance on ecosystem services and non-chemical measures for pest prevention and control and livestock access to open air and roughage feed.

In 2015, over 50.9 million hectares, in 179 countries around the world, were cultivated organically, including areas in conversion [4]. The area under organic management (fully converted and in-conversion) has increased during the last decades in the European Union, where binding standards for organic production have been developed [5, 6]. In the 28 countries forming the EU today, the fraction of organically cultivated land of total agricultural area has been steadily increasing over the last three decades. 0.1%, 0.6%, 3.6%, and 6.2% of agricultural land were organic in 1985, 1995, 2005, and 2015, respectively, equalling 11.2 million ha in 2015 [7–9]. In 7 EU Member States, at least 10% of the agricultural land is organic [7]. In 2003, 125,000 farms in the EU were active in organic agriculture, a number that increased to 185,000 in 2013 [10]. Between 2006 and 2015, the organic retail market has grown by 107% in the EU, to €27.1 billion [7].

This review details the science on the effects of organic food and organic food production on human health and includes

1. studies that directly address such effects in epidemiological studies and clinical trials.
2. animal and in vitro studies that evaluate biological effects of organic compared to conventional feed and food.

Focusing on narrower aspects of production, we then discuss the impact of the production system on

(3) plant protection, pesticide exposure, and effects of pesticides on human health,
(4) plant nutrition, the composition of crops and the relevance for human health,
(5) animal feeding regimens, effects on the composition of animal foods and the relevance for human health.
(6) animal health and well-being, the use of antibiotics in animal production, its role in the development of antibiotic resistance, and consequences of antibiotic resistance for public health.

In the discussion, we widen the perspective from production system to food system and sustainable diets and address the interplay of agricultural production system and individual food choices. The consequences of these aspects on public health are briefly discussed.

Due to a limited evidence base, minimal importance, lack of a plausible link between production system and health, or due to lack of relevance in the European Union, we do not or only briefly touch upon

1. singular food safety events such as outbreaks of diseases that are not clearly caused by the production system (hygiene regulations for plant production and for animal slaughtering and processing are for the most part identical for organic and conventional agriculture) or fraudulent introduction of contaminated feed into the feed market
2. historic events and historic sources of exposure, such as the BSE crisis caused by the now-banned practice of feeding cattle with meat and bone meal from cattle, or continuing effects of the historic use of DDT, now banned in all agricultural contexts globally
3. contaminants from food packaging
4. aspects of food processing, such as food additives
5. the presence of mycotoxins in consequence of post-harvest storage and processing which is governed chiefly by moisture and temperature in storage
6. the use of growth hormones in animal production, which is not permitted in the EU but in several other countries

Furthermore, aspects of environmental sustainability, such as biodiversity and greenhouse gas emissions, may also be affected by the agricultural production system [11, 12] and may affect human health via food security [13, 14]. While these indirect links are outside the scope of this review, we briefly touch on them in the discussion. Also, the focus of this article is on public health, not on occupational health of agricultural workers or local residents, although these issues are considered as part of the epidemiological evidence on pesticide effects. While agricultural standards vary between countries and regions, we maintain a global perspective when appropriate and otherwise focus on the European perspective.

The literature search for this review was carried out at first using the PubMed and Web of Science databases, while applying “organic food” or “organic agriculture” along with the most relevant keywords, through to the end of 2016 (more recent references were included, when relevant, although they were not identified through the systematic search). We made use of existing systematic reviews and meta-analyses when possible. In some cases, where scientific literature is scarce, we included grey literature e.g. from authorities and intergovernmental organisations. We also considered references cited in the sources located.

### Association between organic food consumption and health: Findings from human studies

A growing literature is aiming at characterizing individual lifestyles, motivations and dietary patterns in regard to organic food consumption, which is generally defined from responses obtained from food frequency questionnaires [15–23]. Still, current research on the role of organic food consumption in human health is scarce, as compared to other nutritional epidemiology topics. In particular, long-term interventional studies aiming to identify potential links between organic food consumption and health are lacking, mainly due to high costs. Prospective cohort studies constitute a feasible way of examining such relationships, although compliance assessment is challenging. Considering a lack of biomarkers of exposure, the evaluation of the exposure, i.e. organic food consumption, will necessarily be based on self-reported data that may be prone to measurement error.

Some recent reviews have compiled the findings [24–26] from clinical studies addressing the association between consumption of organic food and health. These studies are scant and generally based on very small populations and short durations, thus limiting statistical power and the possibility to identify long-term effects. Smith-Spangler et al. [25] summarised the evidence from clinical studies that overall no clinically significant differences in biomarkers related to health or to nutritional status between participants consuming organic food compared to controls consuming conventional food. Among studies of nutrient intakes, the OrgTrace cross-over intervention study of 33 males, the plant-based fraction of the diets was produced in controlled field trials, but 12 days of intervention did not reveal any effect of the production system on the overall intake or bioavailability of zinc and copper, or plasma status of carotenoids [27, 28].

In observational studies, a specific challenge is the fact that consumers who regularly buy organic food tend to choose more vegetables, fruit, wholegrain products and less meat, and tend to have overall healthier dietary patterns [18, 29]. Each of these dietary characteristics is associated with a decreased risk for mortality from or incidence of certain chronic diseases [30–36]. Consumers who regularly buy organic food are also more physically active and less likely to smoke [18, 19, 37]. Depending on the outcome of interest, associations between organic vs conventional food consumption and health outcome therefore need to be carefully adjusted for differences in dietary quality and lifestyle factors, and the likely presence of residual confounding needs to be considered. In children, several studies have reported a lower prevalence of allergy and/or atopic disease in families with a lifestyle comprising the preference of organic food [38–44]. However, organic food consumption is part of a broader lifestyle in most of these studies and associated with other lifestyle factors. Thus, in the Koala birth cohort of 2700 mothers and babies from the Netherlands [39], exclusive consumption of organic dairy products during pregnancy and during infancy was associated with a 36% reduction in the risk of eczema at age 2 years. In this cohort, the preference of organic food was associated with a higher content of ruminant fatty acids in breast milk [40], which in turn was associated with a lower odds ratio for parent-reported eczema until age 2y [45].

In the MOBA birth cohort study of 28,000 mothers and their offspring, women reporting a frequent consumption of organic vegetables during pregnancy exhibited a reduction in risk of pre-eclampsia [29] (OR = 0.79, 95% CI 0.62 to 0.99). No significant association was observed for overall organic food consumption, or five other food groups, and pre-eclampsia.

The first prospective study investigating weight change over time according to the level of organic food consumption included 62,000 participants of the NutriNet-Santé study. BMI increase over time was lower among high consumers of organic food compared to low consumers (mean difference as % of baseline BMI = − 0.16, 95% Confidence Interval (CI): −0.32; −0.01). A 31% (95% CI: 18%; 42%) reduction in risk of obesity was observed among high consumers of organic food compared to low consumers. Two separate strategies were chosen to properly adjust for confounders [46]. This paper thus confirms earlier cross-sectional analyses from the same study [18].

In regard to chronic diseases, the number of studies is limited. In the Nutrinet-Santé study, organic food consumers (occasional and regular), as compared to non-consumers, exhibited a lower incidence of hypertension, type 2 diabetes, hypercholesterolemia (in both males and females), and cardiovascular disease (in men) [47] but more frequently declared a history of cancer. Inherent to cross-sectional studies, reverse causation cannot be excluded; for example, a cancer diagnosis by itself may lead to positive dietary changes [48].

Only one prospective cohort study conducted in adults addressed the effect of organic food consumption on cancer incidence. Among 623,080 middle-aged UK women, the association between organic food consumption and the risk of cancer was estimated during a follow-up period of 9.3 y. Participants reported their organic food consumption through a frequency question as never, sometimes, or usually/always. The overall risk of cancer was not associated with organic food consumption, but a significant reduction in risk of non-Hodgkin lymphoma was observed in participants who usually/always consume organic food compared to people who never consume organic food (RR = 0.79, 95% CI: 0.65; 0.96) [37].

In conclusion, the link between organic food consumption and health remains insufficiently documented in epidemiological studies. Thus, well-designed studies characterized by prospective design, long-term duration and sufficient sample size permitting high statistical power are needed. These must include detailed and accurate data especially for exposure assessment concerning dietary consumption and sources (i.e. conventional or organic).

### Experimental in vitro and animal studies

#### In vitro studies

The focus on single plant components in the comparison of crops from organic and conventional production, as discussed further below, disregards the fact that compounds in food do not exist and act separately, but in their natural context [49]. In vitro studies of effects of entire foods in biological systems such as cell lines can therefore potentially point at effects that cannot be predicted from chemical analyses of foods, although a limitation is that most cells in humans are not in direct contact with food or food extracts.

Two studies have investigated the effect of organic and conventional crop cultivation on cancer cell lines, both using crops produced under well-documented agricultural practices and with several agricultural and biological replicates. In the first study extracts from organically grown strawberries exhibited stronger antiproliferative activity against one colon and one breast cancer cell line, compared to the conventionally produced strawberries [50]. In the second study [51] the extracts of organic naturally fermented beetroot juices induced lower levels of early apoptosis and higher levels of late apoptosis and necrosis in a gastric cancer cell line, compared to the conventional extracts. Both studies thus demonstrated notable differences in the biological activity of organic vs. conventionally produced crop extracts in vitro, which should inspire further research. However, neither of these studies allows for the distinction of a selective antiproliferative effect on cancer cells, and general cell toxicity. Therefore it cannot be determined which of the organic or conventional food extracts, if any, had the preferable biological activity in terms of human health.

#### Animal studies of health effects

Considering the difficulties of performing long-term dietary intervention studies in humans, animal studies offer some potential of studying long-term health effects of foods in vivo. However, extrapolation of the results from animal studies to humans is not straight-forward. Studies in this field started almost 100 years ago. A review of a large number of studies [52] concluded that positive effects of organic feed on animal health are possible, but further research is necessary to confirm these findings. Here we focus on the main health aspects.

In one of the best-designed animal studies, the second generation chickens receiving the conventionally grown feed demonstrated a faster growth rate. However, after an immune challenge, chickens receiving organic feed recovered more quickly [53]. This resistance to the challenge has been interpreted as a sign of better health [54, 55].

In one carefully conducted crop production experiment, followed by a rat feeding trial, the production system had an apparent effect on plasma-IgG concentrations but not on other markers of nutritional or immune status [56]. A two-generational rat study based on feed grown in a factorial design (fertilisation x plant protection) of organic and conventional practices revealed that the production system had an effect on several physiological, endocrine and immune parameters in the offspring [57]. Most of the effects identified were related to the fertilisation regimen. None of these studies found that any of the feed production systems was more supportive of animal health.

Several other studies, mostly in rats, have reported some effect of the feed production system on immune system parameters [57–60]. However, the direct relevance of these findings for human health is uncertain.

Collectively, in vitro and animal studies have demonstrated that the crop production system does have an impact on certain aspects of cell life, the immune system, and overall growth and development. However, the direct relevance of these findings for human health is unclear. On the other hand, these studies may provide plausibility to potential effects of conventional and organic foods on human health. Still, most of the outcomes observed in animal studies have not been examined in humans so far.

### Pesticides

#### Plant protection in organic and conventional agriculture

Plant protection in conventional agriculture is largely dependent on the use of synthetic pesticides. Conversely, organic farming generally relies on prevention and biological means for plant protection, such as crop rotation, intercropping, resistant varieties, biological control employing natural enemies, hygiene practices and other measures [61–64]. Yet, certain pesticides are approved for use in organic agriculture. In the EU, pesticides (in this context, more specifically chemical plant-protection products; micro- and macrobiological agents are excluded from this discussion due to their low relevance for human health) are approved after an extensive evaluation, including a range of toxicological tests in animal studies [65]. Acceptable residue concentrations in food are calculated from the same documentation and from the expected concentrations in accordance with approved uses of the pesticides. Currently, 385 substances are authorised as pesticides in the EU (Table 1). Of these*,* 26 are also approved for use in organic agriculture [6, 66] as evaluated in accordance with the same legal framework.

**Table 1:** Active substances approved in the EU and important toxicological properties according to risk assessments by EFSA. Data compiled from the EU pesticides database [66] and from Commission Regulation 889/2008 (consolidated version 2016–11-07) Annex II Sections 1–3 [6]

	Approved in EU agriculture^a^	Also approved in EU organic agriculture^a^
Total number of EU-approved active substances (+ basic substances^b^)	385 (+15)	26 (+10)
Of these:		
Any identified toxicity^c^	340	10
Classified as^d^		
Acutely toxic class 1 + 2 + 3 + 4, total^e^	5 + 17 + 26 + 76, 99	0 + 0 + 2 + 2, 3^f^
Carcinogenicity category 2^g^	27	0
Germ cell mutagenicity category 2^h^	2	0
Reproductive toxicity category 1B + 2^i^	5 + 21	0
Candidate for substitution^j^		
Low ADI/ARfD/AOEL	19	0
Two PBT criteria fulfilled^k^	54	1^l^
Reproductive toxicity 1B^i^	5	0
Endocrine disrupting properties	5	0

^a^Following the practice of [6], the groups of copper compounds, pheromones, fatty acids C7 to C20 (only potassium salts approved for organic agriculture) and paraffin oils are counted as one substance per group. In deviation from [6], plant oils are counted as four substances due to different toxicological properties. Microorganisms (biological plant protection products) are not included

^b^Basic substances are compounds with a low risk profile that are useful in plant protection but primarily have other uses. Basic substances have a different approval procedure compared to active substances in the EU

^c^Identified chronic (ADI – acceptable daily intake assigned) and/or acute toxicity (ARfD – acute reference dose assigned) and/or an identified acceptable operator exposure level (AOEL)

^d^According to Regulation 1272/2008. Only classifications that relate to human health effects and to at least one of the criteria for “candidates for substitution” are included in the table (e.g. skin sensitisation not included). These classifications relate to a compound’s intrinsic hazardous properties, irrespective of its use and exposure pattern. Classifications without any compound are not included in this table (e.g. carcinogenicity class 1 A + B)

^e^Class 1 referring to the highest acute toxicity. Some substances have multiple classifications for different endpoints, therefore the total number of compounds is lower than the sum

^f^Pyrethrins, extract from *Chrysanthemum cinerariaefolium*, are classified as acutely toxic class 4. In addition, two acutely toxic synthetic pyrethroids are approved for use in certain insect traps in organic agriculture: lambda-cyhalothrin (class 3 + 4) and deltamethrin (class 3)

^g^Category 2: “Suspected human carcinogens”. (Category 1A/B: known/presumed to have carcinogenic potential for humans. No substances in this class)

^h^Category 2: “Substances which cause concern for humans owing to the possibility that they may induce heritable mutations in the germ cells of humans”. (Category 1A/B: “Substances known to/to be regarded as if they induce heritable mutations in the germ cells of humans”. No substances in this class)

^i^1B: “Presumed human reproductive toxicant”, 2: “Suspected human reproductive toxicant”. (1A: “Known human reproductive toxicant”. No substances in this class)

^j^Refers to approved substances that should be replaced when less hazardous substances/products are available. The criteria “Carcinogenic 1A/1B” (no compound), “Nature of critical effects” (no compound, no criteria defined) and “Non-active isomers” (two compounds, none approved in organic agriculture) are omitted from this table

^k^PBT criteria: persistent, bioaccumulative and toxic according to criteria specified in [65]

^l^Copper. PBT classification based on accumulation in freshwater/estuarine sediment (P) and toxicity to algae and daphnia (T)

Most of the pesticides approved for organic agriculture are of comparatively low toxicological concern for consumers because they are not associated with any identified toxicity (e.g. spearmint oil, quartz sand), because they are part of a normal diet or constitute human nutrients (e.g. iron, potassium bicarbonate, rapeseed oil) or because they are approved for use in insect traps only and therefore have a negligible risk of entering the food chain (i.e. the synthetic pyrethroids lambda-cyhalothrin and deltamethrin, and pheromones). Two notable exceptions are the pyrethrins and copper. Pyrethrins, a plant extract from *Chrysanthemum cinerariaefolium,* share the same mechanism of action as the synthetic pyrethroid insecticides, but are less stable. Copper is an essential nutrient for plants, animals and humans, although toxic at high intakes and of ecotoxicological concern due to toxicity to aquatic organisms.

Plant protection practices developed in and for organic agriculture may be of benefit to the entire agricultural system [67–70]. This is of specific value for the transition towards sustainable use of pesticides in the EU, which has a strong emphasis on non-chemical plant protection measures including prevention and biological agents [63, 64]. Further, steam treatment of cereal seeds for the prevention of fungal diseases (http://thermoseed.se/) has been developed driven by the needs of organic agriculture as an alternative to chemical seed treatments [71, 72]. These methods are now also being marketed for conventional agriculture, specifically for integrated pest management (IPM) [73].

#### Pesticide use – Exposure of consumers and producers

One main advantage of organic food production is the restricted use of synthetic pesticides [5, 6], which leads to low residue levels in foods and thus lower pesticide exposure for consumers. It also reduces the occupational exposure of farm workers to pesticides and drift exposures of rural populations. On average over the last three available years, EFSA reports pesticide residues below Maximum Residue Levels (MRL) in 43.7% of all and 13.8% of organic food samples. MRLs reflect the approved use of a pesticide rather than the toxicological relevance of the residue. There are no separate MRLs for organic products. A total of 2.8% of all and 0.9% of organic samples exceeded the MRL, which may be due to high residue levels or due to low levels but unapproved use of a particular pesticide on a particular crop [74–76]. Of higher toxicological relevance are risk assessments, i.e. expected exposure in relation to toxicological reference values. On average 1.5% of the samples were calculated to exceed the acute reference dose (ARfD) for any of the considered dietary scenarios, with the organophosphate chlorpyrifos accounting for approximately half of these cases and azole fungicides (imazalil, prochloraz, and thiabendazole) for approximately 15%. None (0%) of the organic samples exceeded the ARfD [74]. Residues of more than one pesticide were found in approximately 25% of the samples but calculations of cumulative risks were not included in the reports [74–76].

The only cumulative chronic risk assessment comparing organic and conventional products known to us has been performed in Sweden. Using the hazard index (HI) method [77], adults consuming 500 g of fruit, vegetables and berries per day in average proportions had a calculated HI of 0.15, 0.021 and 0.0003, under the assumption of imported conventional, domestic conventional, and organic products, respectively [78]. This indicates an at least 70 times lower exposure weighted by toxicity for a diet based on organic foods. There are several routes by which pesticides not approved for use in organic agriculture may contaminate organic products, including spray drift or volatilisation from neighbouring fields, fraudulent use, contamination during transport and storage in vessels or storages where previously conventional products have been contained, and mislabelling by intention or mistake. Overall, however, current systems for the certification and control of organic products ensure a low level of pesticide contamination as indicated by chronic and acute risks above, although they still can be improved [79].

The general population’s exposure to several pesticides can be measured by analysing blood and urine samples, as is routinely done in the US [80] although not yet in Europe. However, a few scattered European studies from France [81–83], Germany [84], the Netherlands [85], Spain [86], Belgium [87], Poland [88] and Denmark [89] have shown that EU citizens are commonly exposed to organophosphate and pyrethroid insecticides. A general observation has been higher urinary concentrations of pesticide metabolites in children compared to adults, most likely reflecting children’s higher food intake in relation to body weight and maybe also more exposure-prone behaviours. The urinary concentrations of generic metabolites of organophosphates (dialkyl phosphates, DAPs) and pyrethroids (3-phenoxybenzoic acid, 3-PBA) found in most of the European studies were similar to or higher than in the US studies. Although urinary metabolite concentration might overestimate the exposure to the parent compounds, due to ingestion of preformed metabolites in food items, several studies have reported associations between urinary metabolite concentrations and neurobehavioral deficits as described below. Besides, the metabolites are not always less toxic than the parent compounds [90].

For the general population, pesticide residues in food constitute the main source of exposure for the general population. This has been illustrated in intervention studies where the urinary excretion of pesticides was markedly reduced after 1 week of limiting consumption to organic food [91–93]. Similar conclusions emerged from studies investigating associations between urinary concentrations of pesticides and questionnaire information on food intake, frequency of different foodstuffs and organic food choices. Thus a high intake of fruit and vegetables is positively correlated with pesticide excretion [94], and frequent consumption of organic produce is associated with lower urinary pesticide concentration [95].

#### Pesticide exposure and health effects

The regulatory risk assessment of pesticides currently practised in the EU is comprehensive, as a large number of toxicological effects are addressed in animal and other experimental studies. Nonetheless, there are concerns that this risk assessment is inadequate at addressing mixed exposures, specifically for carcinogenic effects [96] as well as endocrine-disrupting effects [97, 98] and neurotoxicity [99]. Furthermore, there are concerns that test protocols lag behind independent science [100], studies from independent science are not fully considered [101] and data gaps are accepted too readily [102]. These concerns primarily relate to effects of chronic exposure and to chronic effects of acute exposure, which are generally more difficult to discover than acute effects. Most studies rely on urinary excretion of pesticide metabolites and a common assumption is that the subjects were exposed to the parent chemicals, rather than the metabolites.

The overall health benefits of high fruit and vegetable consumption are well documented [31, 35]. However, as recently indicated for effects on semen quality [103], these benefits might be compromised by the adverse effects of pesticide residues. When benefits are offset by a contaminant, a situation of inverse confounding occurs, which may be very difficult to adjust for [104]. The potential negative effects of dietary pesticide residues on consumer health should of course not be used as an argument for reducing fruit and vegetable consumption. Neither should nutrient contents be used to justify exposures to pesticides. Exposures related to the production of conventional crops (i.e. occupational or drift exposure from spraying) have been related to an increased risk of some diseases including Parkinson’s disease [105–107], type 2 diabetes [108, 109] and certain types of cancers including non-Hodgkin lymphoma [110] and childhood leukaemia or lymphomas, e.g. after occupational exposure during pregnancy [105, 111] or residential use of pesticides during pregnancy [105, 112] or childhood [113]. To which extent these findings also relate to exposures from pesticide residues in food is unclear. However, foetal life and early childhood are especially vulnerable periods for exposure to neurotoxicants and endocrine disruptors. Even brief occupational exposure during the first weeks of pregnancy, before women know they are pregnant, have been related to adverse long-lasting effects on their children’s growth, brain functions and sexual development, in a Danish study on greenhouse worker’s children [114–118].

In order to assess the potential health risk for consumers associated with exposure to dietary pesticides, reliance on epidemiological studies of sensitive health outcomes and their links to exposure measures is needed. Such studies are complicated both by difficult exposure assessment and the necessary long-term follow-up. The main focus so far has been on cognitive deficits in children in relation to their mother’s exposure level to organophosphate insecticides during pregnancy. This line of research is highly appropriate given the known neurotoxicity of many pesticides in laboratory animal models [99] and the substantial vulnerability of the human brain during early development [119].

Most of the human studies have been carried out in the US and have focused on assessing brain functions in children in relation to prenatal organophosphate exposure. In a longitudinal birth cohort study among farmworkers in California (the CHAMACOS cohort), maternal urinary concentrations of organophosphate metabolites in pregnancy were associated with abnormal reflexes in neonates [120], adverse mental development at 2 years of age [121], attention problems at three and a half and 5 years [122], and poorer intellectual development at 7 years [123]. In accordance with this, a birth cohort study from New York reported impaired cognitive development at ages 12 and 24 months and 6 – 9 years related to maternal urine concentrations of organophosphates in pregnancy [124]. In another New York inner-city birth cohort, the concentration of the organophosphate chlorpyrifos in umbilical cord blood was associated with delayed psychomotor and mental development in children in the first 7 years of life [125], poorer working memory and full-scale IQ at 7 years of age [126], structural changes, including decreased cortical thickness, in the brain of the children at school age [127], and mild to moderate tremor in the arms at 11 years of age [128]. Based on these and similar studies, chlorpyrifos has recently been categorised as a human developmental neurotoxicant [129]. Recent reviews of neurodevelopmental effects of organophosphate insecticides in humans conclude that exposure during pregnancy – at levels commonly found in the general population – likely have negative effects on children’s neurodevelopment [130–132]. In agreement with this conclusion, organophosphate pesticides considered to cause endocrine disruption contribute the largest annual health cost within the EU due to human exposures to such compounds, and these costs are primarily due to neurodevelopmental toxicity, as discussed below.

Since growth and functional development of the human brain continues during childhood, the postnatal period is also assumed to be vulnerable to neurotoxic exposures [119]. Accordingly, five-year-old children from the CHAMACOS cohort had higher risk scores for development of attention deficit hyperactive disorder (ADHD) if their urine concentration of organophosphate metabolites was elevated [122]. Based on cross-sectional data from the NHANES data base, the risk of developing ADHD increases by 55% for a ten-fold increase in the urinary concentration of organophosphate metabolites in children aged 8 to 15 years [133]. Also based on the NHANES data, children with detectable concentrations of pyrethroids in their urine are twice as likely to have ADHD compared with those below the detection limit [134]. In addition, associations between urinary concentrations of pyrethroid metabolites in children and parent-reported learning disabilities, ADHD or other behavioural problems in the children have recently been reported in studies from the US and Canada [135, 136].

So far only few prospective studies from the EU addressing associations between urinary levels of pesticides and neurodevelopment in children from the general population have been published. Three studies are based on the PELAGIE cohort in France and present results for organophosphates and pyrethroids respectively [81, 82, 137]. While no adverse effects on cognitive function in six-year-old children were related to maternal urine concentrations of organophosphates during pregnancy, the concentration of pyrethroid metabolites was associated with internalising difficulties in the children at 6 years of age. Also, the children’s own urinary concentrations of pyrethroid metabolites were related to decrements in verbal and memory functions and externalising difficulties and abnormal social behaviour. While this sole European study did not corroborate US birth cohort studies results showing that exposure during pregnancy to organophosphate insecticides at levels found in the general population may harm brain development in the foetus, the exposure levels measured in the PELAGIE cohort were considerably lower for both organophosphates and pyrethroids than those measured in other European studies as well as in studies from the US and Canada. For example, the median urine concentration of organophosphate metabolites in pregnant women in the PELAGIE cohort was 2 – 6 times lower than for pregnant women in other studies [85, 122, 138] and the concentration of the common pyrethroid metabolite 3-PBA was only detectable in urine samples from 30% of the women compared to 80–90% in other studies [88, 139]. Thus, to supplement the French study and the previously mentioned Danish study of greenhouse worker’s children, additional studies that include more representative exposure levels for EU citizens are desirable.

Although exposure levels found in European countries are generally similar to or slightly higher than concentrations found in the US studies, the risk of adverse effects on neurodevelopment in European populations needs to be further characterised. The organophosphate insecticides contributing to the exposure might differ between the US and the EU, also in regard to oral and respiratory intakes. According to the European Food Safety Agency (EFSA), of all the organophosphate insecticides, chlorpyrifos most often exceeds the toxicological reference value (ARfD) [74]. A recent report utilised US data on adverse effects on children’s IQ levels at school age to calculate the approximate costs of organophosphate exposure in the EU. The total number of IQ points lost due to these pesticides was estimated to be 13 million per year, representing a value of about € 125 billion [140], i.e. about 1% of the EU’s gross domestic product. Although there is some uncertainty associated with this calculation, it most likely represents an underestimation, as it focused only on one group of pesticides.

Unfortunately, epidemiological evidence linking pesticide exposure and human health effects is rarely regarded as sufficiently reliable to take into account in the risk assessment conducted by regulatory agencies. For example, the conclusion from the epidemiological studies on chlorpyrifos is that an association of prenatal chlorpyrifos exposure and adverse neurodevelopmental outcomes is likely, but that other neurotoxic agents cannot be ruled out, and that animal studies show adverse effects only at 1000-fold higher exposures [141]. A recent decrease of the maximum residue limit for chlorpyrifos in several crops [142, 143] was based on animal studies only [144], but the limits for the sister compound, chlorpyrifos-methyl were unchanged. This case highlights a major limitation to current approaches to protecting the general population against a broad variety of pesticides.

### Production system and composition of plant foods

Fertilisation in organic agriculture is based on organic fertilisers such as farmyard manure, compost and green fertilisers, while some inorganic mineral fertilisers are used as supplements. Nitrogen (N) input is limited to 170 kg/ha * year [5, 145]. In conventional agriculture, fertilisation is dominated by mineral fertiliser, although farmyard manure is also common in some countries. There is no general limit on N input. Typically, crop yield is limited by plant N availability in organic but not in conventional systems [146] Phosphorus (P) input is on average similar or slightly lower in organic systems [147].

In the absence of particular nutrient deficiency, focusing on single nutrients may be of limited value for evaluating the impact of a food or diet on human health [49]; studies of actual health effects, as discussed above, are generally more informative than studies of single nutrients.

#### Overall crop composition

Metabolomics [148–152], proteomics [153, 154] and transcriptomics [155, 156] studies in controlled field trials provide evidence that the production system has an overall influence on crop development, although there is no direct relevance of these studies for human health. Furthermore, the generally lower crop yield in organic systems [146] as such indicates an effect of management strategy on plant development.

Several systematic reviews and meta-analyses [25, 157–159] with different scopes, inclusion criteria and statistical methods have summarised several hundred original studies reporting some aspect of plant chemical composition in relation to conventional and organic production, in search of overall trends across crops, varieties, soils, climates, production years etc. While the overall conclusions of these systematic reviews look contradictory at first sight, there is agreement between them in most of the detailed findings:

#### Nitrogen and phosphorus

Existing systematic reviews have consistently found lower total nitrogen (7% [157], 10% [159]) and higher phosphorus (standardised mean difference (SMD) 0.82 [25], 8% [157]) in organic compared to conventional crops. These findings lack direct relevance for human health. However, considering the differences in fertilisation strategies discussed above, and the fundamental importance of N, P [160–162], and the N:P ratio [163] for plant development, this may lend some plausibility to other observed effects of the production system on crop composition.

#### Vitamins

Systematic reviews generally agree that the concentration of macronutrients, vitamins, and minerals in crops is either not at all or only slightly affected by the production system. For example, ascorbic acid (vitamin C) has received most attention in this context. Meta-analyses report only small effect sizes of the organic production system on vitamin C content [25, 158, 159].

#### Polyphenols

(Poly)phenolic compounds are not essential nutrients for humans but may play a role in preventing several non-communicable diseases, including cardiovascular disease, neurodegeneration and cancer [164]. The detailed mechanisms are complex and not fully understood [164]. Several environmental and agronomic practices affect the phenolic composition of the crop, including light, temperature, availability of plant nutrients and water management [165]. Under conditions of high nitrogen availability, many plant tissues show a decreased content of phenolic compounds, although there are examples of an opposite relationship [165].

Meta-analyses report modest effect sizes of the production system on total phenolics content, e.g. an increase of 14 – 26% [25, 158, 159]. For some narrower groups of phenolic compounds, larger relative concentration differences (in percent) between organic and conventional crops have been reported [159]. However, such findings represent unweighted averages typically from small and few studies, and are therefore less reliable.

Collectively the published meta-analyses indicate a modestly higher content of phenolic compounds in organic food, but the evidence available does not constitute a sufficient basis for drawing conclusions on positive effects of organic compared to conventional plant products in regard to human health.

#### Cadmium and other toxic metals

Cadmium (Cd) is toxic to the kidneys, can demineralise bones and is carcinogenic [166]. Cd is present naturally in soils, and is also added to soils by P fertilisers and atmospheric deposition. Several factors, including soil structure and soil chemistry, humus content and pH, affect the plant availability of Cd [167]. The application of Cd-containing fertilisers increases Cd concentrations in the crops [167, 168]. Low soil organic matter generally increases the availability of Cd for crops [169], and organically managed farms tend to have higher soil organic matter than conventionally managed farms [11].

The source of Cd in mineral fertilisers is the raw material phosphate rock. The European average Cd content in mineral fertilisers is reported as 68 mg Cd/kg P [170] or 83 mg Cd/kg P [171]. The content of Cd in farmyard manure is variable but apparently in many cases lower: Various types of animal manure in a German collection averaged between 14 and 37 mg Cd/kg P [172].

Smith-Spangler et al. [25] found no significant difference in the Cd content of organic and conventional crops (SMD = −0.14, 95% CI -0.74 – 0.46) in their meta-analysis, while Barański et al. [159] report significantly 48% higher Cd concentration in conventional compared to organic crops (SMD = -1.45, 95% CI -2.52 to −0.39) in another meta-analysis largely based on the same underlying original studies, albeit with different inclusion criteria. We contacted the authors of these meta-analyses in order to understand this discrepancy. An updated version of the Barański meta-analysis, in which some inconsistencies have been addressed and which has been provided by the original authors [173], shows a significant 30% (SMD = −0.56, 95% CI -1.08 to −0.04) elevations of Cd contents in conventional compared to organic crops; in subgroup analysis, this difference is restricted to cereal crops. No updated meta-analysis was available for Smith-Spangler’s analysis [25]; apparently, two large well-designed studies with tendencies towards a lower Cd content in organic crops were not considered [174, 175] although they appear to fulfil the inclusion criteria. Also, a correction for multiple testing has been imposed, which may be overly conservative, given the prior knowledge that mineral fertilisers constitute an important source of Cd to soils and crops. It is unclear how these points would affect the results of Smith-Spangler’s meta-analysis.

There are short-term and long-term effects of Cd influx from fertilisers on the Cd content of crops [167] but no long-term study comparing Cd content in organic and conventional crops is available. In absence of such direct evidence, two long-term experiments indicate a higher slope in Cd concentration over time for minerally fertilised compared to organically fertilised cereal crops [176, 177], after over 100 years of growing.

A lower Cd content of organic crops is therefore plausible due to a lower Cd content in the fertilisers used in organic farming, and potentially due to higher soil organic matter in organic farmland. The general population’s Cd exposure is close to, and in some cases above, the tolerable intake and therefore their exposure to Cd should be reduced. For non-smokers, food is the primary source of exposure, with cereals and vegetables being the most important contributors [168].

For other toxic metals including lead, mercury and arsenic, no differences in concentration in organic and conventional crops have been reported [25, 159]. Uranium (U) is also present as a contaminant of concern in mineral P fertilisers [178], but less so in organic fertilisers [179], and consequently manure-based cropping systems have a lower U load than mineral-fertilised systems at equal P load [179]. Uranium appears to accumulate in mineral-fertilised soils [180], and agricultural activity may increase the U content of surface and groundwater [181, 182]. However, no evidence was found comparing uranium contents of organic and conventional products.

#### Fungal toxins

Regarding fungal toxins in crops, one meta-analysis has reported a lower contamination of organic compared to conventional cereal crops with deoxynivalenol (DON), produced by certain fusarium species [25]. Although not fully understood, fungicide applications may alter fungal communities on cereal leaves, potentially weakening disease-suppressive species [183, 184]. Also, crop rotations including non-cereal crops may contribute to lower infestation with fusarium [185], while N availability is positively associated with cereal DON content [186]. These factors give plausibility to the observed lower DON contamination in organic cereals. In the EU, the mean chronic exposure of toddlers, infants and children to DON is above the tolerable daily intake (TDI), with grains and grain-based products being the main contributors to total exposure. The TDI is based on decreased body weight gain observed in mice [187]. The production system does not have any observed effect on the concentration of ochratoxin A (OTA), another fungal toxin of importance in cereal production [25].

### Animal-based foods

By regulation, herbivores in organic production receive at least 60% of their feed intake as roughage on a dry matter basis. Depending on the seasonal availability of pastures, roughage can be fresh, dried, or silage. Also omnivores in organic production receive roughage as part of their daily feed, and poultry has access to pasture [6]. Corresponding regulations are for the most part missing in conventional animal production. In consequence, feeding strategies in organic animal production include a higher fraction of roughage compared to conventional systems, e.g. for dairy cows [188, 189].

#### Fatty acids

Much of the focus of existing research on compositional differences of organic and conventional animal-based foods is on the fatty acid composition, with a major interest in omega-3 FAs due to their importance for human health. Some studies also address the content of minerals and vitamins.

The FA composition of the feed is a strong determinant of the fatty acid composition of the milk, egg or meat [190, 191]. Grass and red clover, typical roughage feeds, contain between 30% and 50% omega-3 FA of total FA, while the concentrate feeds cereals, soy, corn, and palm kernel cake all contain below 10% omega-3 FA of total FA [190]. Like humans, farm animals turn a small part of dietary alpha-linolenic acid into long-chain omega-3 fatty acids with the help of elongase and desaturase enzymes.

For cow’s milk, a recent meta-analysis reports conclusively an approximately 50% higher content of total omega-3 fatty acids (as percent of total fatty acids) in organic compared to conventional milk [192], generally confirming earlier reviews [25, 189]. Also, the content of ruminant FAs (a group of natural *trans* FAs produced in the cow’s rumen) is higher in organic milk. The content of saturated fatty acids, mono-unsaturated fatty acids and omega-6 PUFA was similar in organic and conventional milk [192].

A considerable statistical heterogeneity in these findings is reported. Individual differences described above are based on results from between 11 and 19 included studies. The observed differences are plausible, because they are directly linked to differences in feeding regimens. It should also be noted that several other factors influence the fatty acid composition in milk [193]. Specifically, the season (indoor vs. outdoor) has an impact on the feeding regime [188] and therefore on the omega-3 content of milk. However, the content of omega-3 fatty acids is higher in organic milk during both the outdoor and indoor seasons [189].

For eggs, it is likewise well described that the FA composition of the feed [190] and consequently the access to pasture [194, 195] such as in organic systems, is a strong determinant of the fatty acid composition of the egg. However, only few studies have compared the FA composition in organic and conventional eggs [196] and a systematic review is not available. A higher omega-3 content of organic eggs is plausible but has not been documented.

A total of 67 original studies report compositional aspects of meat (mainly beef, chicken, lamb, and pork) from organic and conventional husbandry and were recently summarised in a meta-analysis [197]. Based on 23 and 21 studies respectively, the content of total PUFA and omega-3 PUFA was found to be significantly higher (23 and 47%, respectively) in organic compared to conventional meats. Weighted by average consumption in Europe, choosing organic instead of conventional meat, while maintaining a constant consumption, increased the intake of PUFA and omega-3 FA from meat by 17 and 22%, respectively [198]. These findings are plausible, especially in the case of omega-3 PUFA, considering the known differences in feeding regimens in organic and conventional production. However, few studies were available for each analysis, leaving many analyses with high uncertainty and poor statistical power. Furthermore, fatty acid metabolism differs between ruminants and monogastric animals [190]. Also, the actual differences in feeding regimens between conventionally and organically raised animals may differ by species, and by country. The variation between studies and between species was large, and the overall reliability of these results is therefore lower compared to milk above. This meta-analysis therefore indicates a plausible increase in omega-3 contents in organic meats, but more well-designed studies are needed to confirm this effect [197].

Dairy products account for 4–5% of the total PUFA intake in most European populations, while meat and meat products contribute another 7–23% [199]. The contribution of milk fat to omega-3 PUFA intake (approximated as intake of α-linolenic acid) has been estimated at 5–16% [200, 201], while meat contributes with 12–17% [201, 202]. The effect of exchanging organic for conventional dairy products on omega-3 PUFA intake while maintaining a constant consumption has not been examined rigorously. From the intake and composition data presented here, it can be estimated that choosing organic products would increase the average dietary omega-3 PUFA intake by 2.5–8% (dairy) and by a less certain 2.5–4% (meat). A recent preliminary estimate based on FAO food supply data resulted in similar numbers [198]. For certain population groups and fatty acids, these numbers could be higher, and an increased omega-3 PUFA consumption is generally desirable, as some subpopulations have a lower-than-recommended intake of omega-3 PUFA [203]. However, overall, the effect of the animal production system on omega-3 PUFA intake is minor, and no specific health benefits can be derived. Furthermore, other dietary omega-3 PUFA sources, specifically certain plant oils and fish, are available that carry additional benefits [204–206]. The existence of specific health benefits of ruminant *trans* fatty acids (as opposed to industrial *trans* fatty acids) is indicated by some studies [207] but not strongly supported [208]. Taking into account the actually consumed amounts of ruminant *trans* fatty acids, this is likely lacking public health relevance [208].

#### Trace elements and vitamins

A recent meta-analysis points to a significantly higher content of iodine (74%) and selenium (21%) in conventional milk and of iron (20%) and tocopherol (13%) in organic milk based on six, four, eight and nine studies respectively [192]. Iodine deficiency during pregnancy and infancy leads to impairment of brain development in the offspring, while excess iodine intake is associated with similar effects, and the window of optimal iodine intake is relatively narrow [209]. Overall, iodine intake in Europe is low and mild deficiency is prevalent [210]. The preferred way of correcting deficiency is salt iodisation [210, 211], because salt is consumed almost universally and with little seasonal variation [212].

Feed iodine supplementation is not linked by regulation to the production system in the EU, as iodine is listed as approved feed additive, and the maximum amount of supplementation is the same for all milk production. Optimum dairy cow supplementation should be seen in relation to other national strategies for human iodine intake. This should also take into account human subpopulations with low or no intake of dairy products.

For tocopherol, selenium and iron, a higher content is generally desirable, and in the case of selenium milk is an important source. However, the concentration differences between organic and conventional milk are modest and based on a few studies only.

### Antibiotic resistant bacteria

Overly prevalent prophylactic use of antibiotics in animal production is an important factor contributing to increasing human health problems due to resistant bacteria. Antibiotic use is strongly restricted in organic husbandry, which instead aims to provide good animal welfare and enough space in order to promote good animal health.

Antibiotics constitute an integral part of intensive animal production today, and farm animals may act as important reservoirs of resistant genes in bacteria [213, 214]. It is reported that a substantial proportion (50 – 80%) of antibiotics are used for livestock production worldwide [215]. On a “per kg biomass” basis, in 2014, the amount of antimicrobial drugs consumed by farm animals was slightly higher than the antimicrobial drugs used for humans in the 28 EU/EEA countries surveyed, with substantial differences between countries regarding volumes and types of substances [216].

In recent decades, there have been increasing concerns that the use of antibiotics in livestock would contribute to impairing the efficiency of antibiotic treatment in human medical care [217]. Despite the lack of detailed information on transmission routes for the vast flora of antibiotic-resistant bacteria and resistance genes, there is a global need for action to reduce the emerging challenges associated with the reduced efficiency of antibiotics and its consequences for public health, as well as for the environment more generally [218, 219].

The use of antibiotics may increase the economic outcome of animal production [220, 221], but the spreading of multi-resistant genes is not just a problem for the animal production sector alone. Negative effects are affecting parts of society not directly associated with livestock production. This means that the costs of side effects are borne by society in general and not primarily by the agricultural sector. However, the generalisation cannot be made that all antibiotic treatment in farm animals represents a hazard to public health [222, 223].

The use of antibiotics in intensive livestock production is closely linked to the housing and rearing conditions of farm animals. Specific conditions for conventional livestock farming in different countries, as well as farmers’ attitudes, may differ between countries, e.g. conventional pig production at above EU animal welfare standards and farmers’ attitudes in Sweden [224, 225]. Conventional production is typically aiming for high production levels with restricted input resources such as space, feed etc., and these conditions may cause stress in the individual animal as it is unable to cope with the situation, e.g. in pig production [226, 227]. This means that higher stocking density, restricted space and barren environment are factors increasing the risk of the development of diseases, and therefore it is more likely that animals under these conditions need antibiotic treatments.

Organic production aims for less intensive animal production, which generally means that the animals have access to a more spacious and enriched environment, access to an outdoor range and restricted group sizes, and other preconditions [70]. This would ultimately decrease the need for preventive medication of the animals as they can perform more natural behaviours and have more opportunity to maintain a good health. However, in practice, the health status of organic livestock is complex and disease prevention needs to be adapted to the individual farm [228]. A report on the consequences of organic production in Denmark demonstrates that meeting the requirements of organic production has several positive consequences in relation to animal welfare and health [70].

According to EU regulations, routine prophylactic medication of animals in organic production is not allowed. However, diseases should be treated immediately to avoid suffering, and the therapeutic use of antibiotics is allowed, but with longer withdrawal periods than in conventional production [5]. Furthermore, products from animals treated more than three times during 12 months, or, if their productive lifecycle is less than 1 year, more than once, cannot be sold as organic [6]. This means that therapeutically the same antibiotics used in conventional farming may be used in organic farming, but under different conditions. For example, antibiotics mainly used for sub-therapeutic treatment as prophylaxis are never considered in organic production.

While the organic regulations aim for a low use of antibiotics in livestock production, the actual use of antibiotic drugs in European organic compared to conventional animal husbandry is not comprehensively documented. Scattered studies indicate that the antibiotic use generally is substantially higher in conventional compared to organic systems, especially for pigs (approximately 5 – 15-fold higher) [229, 230]. In studies from Denmark [231] and the Netherlands [232], the antibiotic use in dairy cows was 50% and 300% higher in conventional compared to organic systems, although a Swedish study found no differences in disease treatment strategies between organic and conventional dairy farms, e.g. for mastitis [233]. While only sparingly documented (e.g. [234, 235]), there is only little use of antibiotics in EU organic broiler production. This is a consequence of regulations prohibiting prophylactic use and prescribing long withdrawal periods before slaughter [6, 236], in conjunction with the fact that it is not feasible to treat single animals in broiler flocks. In conventional broiler production, antibiotic use is common (e.g. [237–239]).

Recently, gene sequencing has revealed that the routes of transmission of resistance genes between human and farm animal reservoirs seem to be complex [213, 222, 240]. Nevertheless, a recent EFSA report found that “in both humans and animals, positive associations between consumption of antimicrobials and the corresponding resistance in bacteria were observed for most of the combinations investigated” [241], which has subsequently been strengthened [216]. In addition to direct transmission between animals and humans via contact or via food, resistant strains and resistance genes may also spread into the environment [242].

Previously, it has been postulated that a reduced need and use of antibiotics in organic livestock production will diminish the risk of development of antibiotic resistance [243], and this has also been demonstrated with regard to resistant *E. coli* in organic pigs compared to conventional pigs [244]. It has also been shown that the withdrawal of prophylactic use of antibiotics when poultry farms are converted from conventional to organic production standards leads to a decrease in the prevalence of antibiotic-resistant *Salmonella* [245].

Resistant bacteria may be transferred within the production chain from farm to fork [246]. It has been found that organic livestock products are less likely to harbour resistant bacteria in pork and chicken meat [25].

In pig production, particular attention has been paid to methicillin-resistant *Staphylococcus aureus* (MRSA), and in Dutch and German studies, for example, MRSA has been isolated in 30 and 55% respectively of all pigs tested [247, 248]. Furthermore, it has been found that healthy French pig farmers are more likely to carry MRSA than control persons [249] and that they carry similar strains of MRSA to those found on their pig farms [250]. However, the prevalence of MRSA in pig production may differ between conventional and organic farms, and in a meta-study in 400 German fattening pig herds, the odds ratio (OR) for MRSA prevalence was 0.15 (95% CI 0.04, 0.55) in organic (*n* = 23) compared to conventional (*n* = 373) pig farms [248]. Multivariate adjustment for potential risk factors rendered this association non-significant, suggesting that it was carried by other factors, including factors that are regulated in or associated with organic production, such as non-slatted floors, no use of antibiotics, and farrow-to-finish herd types. Furthermore, even if there are considerable differences in antibiotic use between countries, it has been found that antibiotic resistance is less common in organic pigs compared to conventional pigs in France, Italy, Denmark, and Sweden [251, 252].

Although it is rare for conventional farms to adopt knowledge about management and housing from organic production except when converting farms in line with organic standards, there may be options to improve animal health and welfare by knowledge transfer to conventional farms in order to reduce the use of antibiotics [253].

Within organic production, labelling requires full traceability in all steps in order to guarantee the origin of the organic products being marketed [5]. Application of the general principle of organic regulations about transparency throughout the food chain can be used to mitigate emerging problems of transmission of antimicrobial resistance. However, transition to organic production for the whole livestock sector would, on its own, be only part of a solution to the antibiotics resistance issue, because factors outside animal production, such as their use in humans, will be unaffected.

## Discussion

An assessment of the human health effects associated with diets based on organic food production must rely on two sets of evidence. The first set of evidence is the epidemiological studies comparing population groups with dietary habits that differ substantially in regard to choices of organic v. conventional products. These studies are to some extent complemented by experimental studies using animal models and in vitro models. The second set of data relies on indirect evidence such as chemical analyses of food products and their contents of nutrients and contaminants or antibiotic use and resistance patterns, in onsequence of agricultural production methods. Both sets of results are associated with certain strengths and weaknesses.

The few human studies that have directly investigated the effects of organic food on human health have so far yielded some observations, including indications of a lower risk of childhood allergies, adult overweight/obesity [18, 46] and non-Hodgkin lymphoma (but not for total cancer) [37] in consumers of organic food. Owing to the scarcity or lack of prospective studies and the lack of mechanistic evidence, it is presently not possible to determine whether organic food plays a causal role in these observations. However, it has also been observed that consumers who prefer organic food have healthier dietary patterns overall, including a higher consumption of fruit, vegetables, whole grains, and legumes and a lower consumption of meat [18, 29, 37]. This leads to some methodological difficulties in separating the potential effect of organic food preference from the potential effect of other associated lifestyle factors, due to residual confounding or unmeasured confounders. These dietary patterns have in other contexts been associated with a decreased risk of several chronic diseases, including diabetes and cardiovascular disease [30–36]. It is therefore expected that consumers who regularly eat organic food have a decreased risk of these diseases compared to people consuming conventionally-produced food, as a consequence of dietary patterns. These dietary patterns appear also to be more environmentally sustainable than average diets [254].

Food analyses tend to support the notion that organic foods may have some health benefits. Consumers of organic food have a comparatively low dietary exposure to pesticides. Although chemical pesticides undergo a comprehensive risk assessment before market release in the EU, there are important gaps in this risk assessment. In some cases, specifically for cognitive development during childhood as an effect of organophosphate insecticide exposure during pregnancy, epidemiological studies provide evidence of adverse effects [140, 255]. Organic agriculture allows for lower pesticide residues in food and may be instrumental in conventional agriculture’s transition towards integrated pest management by providing a large-scale laboratory for non-chemical plant protection.

This review emphasizes that pesticide exposure from conventional food production constitutes a main health concern. A key issue that has only recently been explored in biomedical research is that early-life exposure is of major concern, especially prenatal exposure that may harm brain development. Most insecticides are designed to be toxic to the insect nervous system, but many higher species depend on similar neurochemical processes and may therefore all be vulnerable to these substances [129]. Besides insecticides, experimental studies suggest a potential for adverse effects on the nervous system for many herbicides and fungicides as well [99]. However, no systematic testing is available since testing for neurotoxicity – especially developmental neurotoxicity – has not consistently been required as part of the registration process, and allowable exposures may therefore not protect against such effects. At least 100 different pesticides are known to cause adverse neurological effects in adults [129], and all of these substances must therefore be suspected of being capable of damaging also developing brains. The need for prevention of these adverse outcomes is illustrated by the recent cost calculations [140] and the additional risk that pesticide exposures may lead to important diseases, such as Parkinson’s disease, diabetes and certain types of cancer.

The outcomes in children and adults and the dose-dependences are still incompletely documented, but an additional limitation is the lack of exposure assessments in different populations and also their association with dietary habits. The costs from pesticide use in regard to human health and associated costs to society are likely to be greatly underestimated due to hidden and external costs, as recently reviewed [256]. Also, gaps in the regulatory approval process of pesticides may lead to important effects being disregarded and remaining undetected.

In regard to nutrients, organic dairy products, and probably also meat, have an approximately 50% higher content of omega-3 fatty acids compared to conventional products. However, as these products only are a minor source of omega-3 fatty acids in the average diet, the nutritional significance of this effect is probably low (although this has not been proven). The nutritional content of crops is largely unaffected by the production system, according to current knowledge. Vitamins and minerals are found in similar concentrations in crops from both systems. One exception is the increased content of phenolic compounds found in organic crops, although this is still subject to uncertainty despite a large number of studies that have addressed this issue. Accordingly, although in general being favourable for organic products, the established nutritional differences between organic and conventional foods are small, and strong conclusions for human health cannot currently be drawn from these differences. There are indications that organic crops contain less cadmium compared to conventional crops. This is plausible, primarily because mineral fertiliser is an important source of cadmium in soils. However, notably, long-term farm pairing studies or field trials that are required for definitely establishing or disproving this relationship are lacking. Owing to the high relevance of cadmium in food for human health, this lack of research constitutes an important knowledge gap.

With respect to the development of antibiotic resistance in bacteria, organic animal production may offer a way of restricting the risks posed by intensive production, and even decreasing the prevalence of antibiotic resistance. Organic farm animals are less likely to develop certain diseases related to intensive production compared to animals on conventional farms. As a consequence, less antibiotics for treating clinical diseases are required under organic management, where their prophylactic use also is strongly restricted. This decreases the risk for development of antibiotic resistance in bacteria. Furthermore, the transparency in organic production may be useful for acquiring knowledge and methods to combat the rising issues around transmission of antimicrobial resistance within food production.

It appears essential that use of antibiotics in animal production decreases strongly or completely ceases in order to decrease the risk of entering a post-antibiotic era. The development and upscaling of rearing systems free or low in antibiotic use, such as organic broiler production, may be an important contribution of organic agriculture to a future sustainable food system.

Most of the studies considered in this review have investigated the effects of agricultural production on product composition or health. Far less attention has been paid to the potential effects of food processing. Processing may affect the composition of foods and the bioavailability of food constituents. It is regulated [5] and recognised [257] that food additives are restricted for organic products compared to conventional products. It is also recognised that the degree of food processing may be of relevance to human health [258, 259]. In organic food processing, the processing should be done “with care, preferably with the use of biological, mechanical and physical methods” [5] but there are no specific restrictions or guidelines. With the exception of chemical additives, it is unknown whether certain food processing methods (e.g. fermentation of vegetables, pasteurisation of vegetables) are more prevalent in organic or conventional products or consumption patterns, or whether such differences are of relevance to human health.

The scopes of two recent reports, from Norway [260] and Denmark [70], in part overlap with the present work. Broadly, the reviewed results and conclusions presented in those reports are in line with this article. For several topics, important new evidence has been published in recent years. Consequently, in some cases stronger conclusions can be drawn today. Furthermore, the present review includes epidemiological studies of pesticide effects in the evidence base reviewed.

Over all, the evidence available suggested some clear and some potential advantages associated with organic foods. The advantages in general do not necessarily require organic food production as strictly defined in current legislation. Certain production methods, such as changes in the use of pesticides and antibiotics, can be implemented in conventional production, e.g. supporting a development towards a sustainable use of pesticides [261]. Thereby, practices and developments in organic agriculture can have substantial public health benefits also outside the organic sector.

Diet choices and the associated food production methods also have important impacts on environmental sustainability [254]. Consumption patterns of consumers preferring organic food [16, 18, 19, 37, 47] seem to align well with sustainable diets [2]. These consumption patterns also show some similarities with the Mediterranean Diet [262–265] and with the New Nordic Diet [266–269], with lower dietary footprints in regard to land use, energy and water consumption, and greenhouse gas emissions compared to concurrent average diets. Further evaluation is needed to assess the extent to which organic food systems can serve as example of a sustainable food systems [270].

For the development of healthy and environmentally-sustainable food systems in the future, production and consumption need to be considered in an integrated manner [2, 271]. While an evaluation of overall impacts of different food systems on environmental sustainability would be highly desirable [270], the present review has attempted to assess the human health issues in regard to organic production methods and consumer preferences for organic food, both important aspects of sustainability.

## Conclusions

Suggestive evidence indicates that organic food consumption may reduce the risk of allergic disease and of overweight and obesity, but residual confounding is likely, as consumers of organic food tend to have healthier lifestyles overall. Animal experiments suggest that growth and development is affected by the feed type when comparing identically composed feed from organic or conventional production. In organic agriculture, the use of pesticides is restricted, and residues in conventional fruits and vegetables constitute the main source of human exposures. Epidemiological studies have reported adverse effects of certain pesticides on children’s cognitive development at current levels of exposure, but these data have so far not been applied in the formal risk assessments of individual pesticides. The nutrient composition differs only minimally between organic and conventional crops, with modestly higher contents of phenolic compounds in organic fruit and vegetables. There is likely also a lower cadmium content in organic cereal crops. Organic dairy products, and perhaps also meats, have a higher content of omega-3 fatty acids compared to conventional products, although this difference is of likely of marginal nutritional significance. Of greater concern is the prevalent use of antibiotics in conventional animal production as a key driver of antibiotic resistance in society; antibiotic use is less intensive in organic production. Thus, organic food production has several documented and potential benefits for human health, and wider application of these production methods also in conventional agriculture, e.g., in integrated pest management, would therefore most likely benefit human health.

## Abbreviations

3-PBA: 3-phenoxybenzoic acid
ADHD: Attention deficit hyperactivity disorder
ADI: Acceptable daily intake
AOEL: Acceptable operator exposure level
ARfD: Acute reference dose
BMI: Body mass index
BSE: Bovine spongiform encephalopathy
Cd: Cadmium
CHAMACOS: Center for the health assessment of mothers and children of Salinas
CI: Confidence interval
DAP: Dialkyl phosphate
DDT: Dichlorodiphenyltrichloroethane
DON: Deoxynivalenol
*E. coli*: *Escherichia coli*
EEA: European Economic Area
EFSA: European Food Safety Authority
EU: European Union
FA: Fatty acid
FAO: Food and Agriculture Organization of the United Nations
ha: Hectare
HI: Hazard index
IgG: Immunoglobulin G
IPM: Integrated pest management
IQ: Intelligence quotient
MRL: Maximum residue level
MRSA: Methicillin-resistant *Staphylococcus aureus*
N: Nitrogen
NHANES: National health and nutrition examination survey
OR: Odds ratio
OTA: Ochratoxin A
P: Phosphorus
PBT: Persistent, bioaccumulative, toxic
PELAGIE: Perturbateurs endocriniens: étude longitudinale sur les anomalies de la grossesse, l’infertilité et l’enfance (endocrine disruptors: longitudinal study on disorders of pregnancy, infertility and children)
PUFA: Polyunsaturated fatty acid
RR: Relative risk
SMD: Standardized mean difference
TDI: Tolerable daily intake
U: Uranium
UK: United Kingdom
US: United States
